# A Survey of Breastfeeding Attitudes and Health Locus of Control in the Nigerian Population

**DOI:** 10.1007/s10995-023-03638-z

**Published:** 2023-04-08

**Authors:** Adenike Adegbayi, Andrew Scally, Valerie Lesk, Barbara J Stewart-Knox

**Affiliations:** 1 Department of Psychology, University of Bradford, Richmond Road, BD71DP Bradford, UK; 2 School of Clinical Therapies, University College Cork, Cork, Ireland

**Keywords:** Breastfeeding, Iowa Infant Feeding Attitude Scale, Attitudes, Health locus of control, Nigeria, Survey

## Abstract

**Objectives:**

Breastfeeding is important to infant health and survival in sub-Saharan Africa. To promote breastfeeding effectively, understanding of psychological factors associated with infant feeding choices is required. This study investigated breastfeeding attitudes and health locus of control (HLoC) in a Nigerian community sample.

**Methods:**

Men and women (N = 400) (71% female; mean age 34.2 years/ range 18–86 years) were recruited through community groups in Nigeria. Self-report survey by questionnaire measured breastfeeding attitudes using the Iowa Infant Feeding Attitude Scale (IIFAS) and health locus of control using the Multidimensional Health Locus of Control Scale (MHLoCs).

**Results Mean:**

IIFAS scores (mean = 57.7; sd = 7.8) became less favourable with increasing age (p = 0.02). Men had higher IIFAS scores (mean = 58.6; sd = 7.6) than women (mean = 56.6; sd = 8.0) indicating more favourable attitudes toward breastfeeding (p = 0.02). Women scored higher than men on external chance HLoC (ECHLoC) (p = 0.003) and external powerful others HLoC (EPHLoC) (p = 0.02). Increasing age was associated with higher scores on ECHLoC (p < 0.01) and EPHLoC (p < 0.01). Multiple linear regression analysis was significant (p < 0.001) and explained 7.8% of variance in breastfeeding attitude. Lower IIFAS scores, reflecting more negative attitudes to breastfeeding, were associated with higher ECHLoC (p < 0.01) and EPOHLoC (p < 0.05). Higher IIFAS scores, reflecting more positive attitudes to breastfeeding, were associated with greater IHLoC (p < 0.01). Neither age nor gender were associated with IIFAS scores in the final model.

**Conclusions:**

This implies a need to explore health locus of control when promoting positive attitudes to breastfeeding and supporting families in breastfeeding advocacy.

## Significance

*What is already known?* Infant feeding decisions are made in a social context and influenced by significant others. Yet, most studies of breastfeeding attitudes have exclusively considered women of childbearing age and mostly in Western cultures.

*What this study adds?* This study meets a need to understand societal attitudes to breastfeeding in diverse cultures. The research also tests the theory that health-related locus of control (H-RLoC) will be related to breastfeeding attitudes. Knowledge of societal attitudes toward breastfeeding and of HRLoC will help to inform effective breastfeeding promotions that are tailored to the specific needs of Nigerian people.

## Introduction

Infant feeding attitude is an important predictor of infant feeding decisions (Lau et al., 2017; Scott et al., 2004), a determinant of adherence to infant feeding guidelines (Li Huang et al., 2021; Hamze et al., 2019; Alkusayer et al., 2018) and predictive of duration and exclusivity of breastfeeding (Dadzie et al., 2023; Li et al., 2021; Yu et al., 2020; Donnan et al., 2013). To promote breastfeeding effectively, therefore, it is imperative to understand attitudes toward breastfeeding. The Iowa Infant Feeding Attitude Scale (IIFAS) was developed by de la Mora et al. (1999) to measure breastfeeding attitudes for which it has since been employed in various countries (Jefferson, 2017; Karande & Peker, 2012; Ahmed & El Guindy, 2011; Ho & McGrath, 2011; Sittlington et al., 2007; Simmie, 2006; de la Mora, et al., 1999). Responses to the IOWA infant feeding attitude scale (IIFAS) have been shown to predict infant feeding intention (Alkusayer et al., 2018; Chang et al., 2012; Sittlington et al., 2007; Dungy et al., 1999), chosen infant feeding method (Chen et al., 2013) and duration of breastfeeding (Alkusayer et al., 2018; Holbrook et al., 2013; Niela-Vilen et al., 2016). Relatively few studies (e.g. Jefferson, 2017; Lau et al. 2017; van Wagenen et al., 2015; Mitchell-Box et al., 2013; Chang et al., 2012) appear to have surveyed breastfeeding attitudes in population samples that include both men and women of a range of age.

The locus of control concept originates from Rotter’s (1966) social learning theory which posits that reinforcement for a particular behaviour is dependent upon whether perceived control over that behaviour is internally or externally located. Internal reinforcement for a behaviour occurs when an individual believes that the behaviour is within their control (internal locus of control) and external reinforcement occurs when an individual believes their behaviour to be dependent on factors external to them such as fate or chance (external locus of control) (Ayan & Eser, 2016). Levenson (1973) extended the locus of control concept by proposing a three-factor model by expanding the external locus of control to include two dimensions, chance and powerful others. Health locus of control (HLoC) refers to an individual’s beliefs about control over their health (Walston et al. 1978). A high external health locus of control (EHLoC) is the belief that other people such as family members and health providers or chance/fate determine health outcomes. A high internal health locus of control (IHLoC) suggests that an individual believes that their own actions determine their health outcomes. The Multidimensional Health Locus of Control Scale (MHLC) (Wallston et al., 1978) was developed to assess health related locus of control in healthy and non-healthy populations. People high on IHLoC are more likely to take responsibility for their health and to engage in healthy behaviour (Davey et al., 2019). Previous studies are of the consensus that individuals with high IHLoC are more likely to actively engage in dietary health promoting behaviour (Jang and Beck, 2018; Poínhos et al., 2014; Rongen et al., 2014; Helmer et al., 2012; Grotz et al., 2011; Wallston 2005; Steptoe and Wardle 2001; Springer et al., 1994). The evidence appears inconsistent, however, in that two recent studies found no association between dimensions of HLoC and dietary quality (Stewart-Knox et al., 2021; Hildt-Ciupińska & Pawłowska-Cyprysiak, 2020). A survey in Nigeria (N = 291) reported that women with higher IHLoC were more confident about breastfeeding (Lawal & Idemudia, 2017).

The World Health Organization (WHO) recommends that infants be exclusively breastfed for the first six months of life with continued breastfeeding up to 24 months (WHO, 2018). Exclusive breastfeeding rates in Nigeria, although improved in recent years, remain well below recommended levels (Agho et al., 2019; Ogbo et al., 2015; United Nations Children’s Fund (UNICEF), 2019; Balogun et al., 2017; National Bureau of Statistics (NBS), 2017; Adewuyi & Adefemi, 2016; Lawan et al., 2014). Instruments with established psychometric validity for measuring breastfeeding attitudes have originated in Western countries (McRae, 2019; Iliadou et al., 2019; Reyes et al., 2019). Findings from these locations may not be applicable to Nigeria, where breastfeeding is deeply enshrined in cultural and religious practices (Adepoju et al., 2019; Leshi et al., 2016; Akinremi & Samuel, 2015). Understanding factors driving breastfeeding attitudes is imperative so that families in Nigeria can be effectively supported in exclusively breastfeeding during the first six months postnatally. Breastfeeding occurs in a social context. To enable exclusive breastfeeding as recommended for the first six months of life (WHO 2018), therefore, it is important to understand attitudes to breastfeeding in the wider community with a view to promoting a breastfeeding friendly society. Previous research has suggested that decisions about breastfeeding are made prior to conception (Giles et al., 2015; Chang et al., 2012). Yet most studies on breastfeeding attitudes have been conducted exclusively on childbearing women. Existing literature implies that although breastfeeding attitudes tend to be more positive in women (Jefferson, 2017; Chang et al., 2012), that partners and other family members exert considerable influence over infant feeding decisions (Satilmis et al., 2022; Vazquez-Vazquez, et al., 2022; Joseph & Earland, 2019; Ogundele et al., 2019; Cox et al. 2015; Chezem 2012; Michell-Box et al. 2013; Reid et al. 2010; Shaker et al., 2004).

The few existing studies of breastfeeding conducted in Nigeria (Akinremi & Samuel, 2015) found that although most respondents reported positive attitudes to breastfeeding, more than half (63.8%) did not exclusively breastfeed for the first six months of life. There is also evidence that younger women in Nigeria are less likely than older women to breastfeed exclusively for six months (Odukoya et al., 2022; Ogundare et al., 2021; Olasinde et al., 2021; Emmanuel & Clow, 2020). Initiatives to improve breastfeeding attitudes have been shown to lengthen the duration of exclusive breastfeeding (Li et al., 2021). People are also likely to vary in the degree to which breastfeeding attitudes are influenced by others (Satilmis et al., 2022; Vazquez-Vazquez, et al., 2022) and/or attributed to chance (Springer et al., 1994). Health locus of control can be assumed to be related to the independence people exert over breastfeeding attitudes and practices (Davey et al., 2019). Understanding how an individual’s HLoC relates to breastfeeding attitudes in a Nigerian community sample hold potential for promotion of exclusive breastfeeding to be tailored to individual and societal health loci of control (Marton et al., 2020; Davey et al., 2019; Jang & Baek, 2018). The current study therefore surveyed breastfeeding attitudes and HLoC across gender, age and society. Given results from previous research, we predict that people who hold an IHLoC orientation will have more positive attitudes to breastfeeding and that people who hold an EHLoC will have less positive attitudes to breastfeeding.

## Method

### Sampling and Procedure

The study was conducted in Nigeria in accordance with the ethical standards laid down in the 1964 Declaration of Helsinki and later amendments made prior to the time of data collection. Ethical approval was granted by the University of Bradford Humanities, Social and Health Sciences Research Panel.

A survey design was employed for which a convenience sample (N = 427) of men and women was recruited from among the population through direct communication (n = 200), formal and informal community groups and artisan communities (n = 177) with subsequent snowballing (n = 50). Data were collected over a six-month period during 2018 in four major locations across Nigeria (Lagos, Kaduna, Abuja and Osun). These locations were chosen because they serve as administrative and commercial centres and owing to rural-urban drift, they constitute a ‘melting pot’ for people of different cultures residing in in Nigeria (Ajaero and Onokala, 2013). These locations, therefore, had potential to recruit participants across different socio-cultural and demographic characteristics. Inclusion criteria were being 18 years of age or above, with no history of a mental health condition and fluent in English. Given the objective was to obtain a societal sample derived from the broader community, both men and women of a range of age, and including parents and non-parents were recruited. Because the opinions and views of broader society were sought, no attempt was made to control for experience of breast feeding.

Potential participants were assured of confidentiality and that no identifying information would be collected. No incentives were offered. Following a 24-hour cancellation period between recruitment and participation 20 volunteers did not attend to participate. Prior to data collection, participants were encouraged to read an information sheet detailing the purpose of the study, explaining how data would be collected and used, and what would be required of them should they agree to participate. After receiving information and having provided written consent to take part, participants completed the questionnaire on paper in a location of their choosing and in the presence of the researcher. Upon completion, the researcher provided a full debrief.

### Materials

The survey tool included sociodemographic questions that enquired of gender, age, education level, employment, relationship status and whether respondents had children.

#### Iowa Infant Feeding Attitude Scale

Attitudes towards breastfeeding were measured by the 17-item Iowa Infant Feeding Attitude Scale (IIFAS) developed by de la Mora at al. (1999). The IIFAS has been used to determine breastfeeding attitudes in various countries and cultures (Jefferson, 2017; Karande & Peker, 2012; Ahmed & El Guindy, 2011; Ho & McGrath, 2011; Sittlington et al., 2007; Simmie, 2006; de la Mora, et al., 1999). The scale measures breastfeeding attitudes as a single factor within which half of the items are worded in a manner favourable to breastfeeding and the other half favourable to formula feeding. Responses were on a Likert scale ranging from one (strong disagreement) to five (strong agreement). Items favouring formula feeding were reverse-scored before a total score was computed. Scores ranged from 17 to 85 with higher scores indicating more positive attitudes to breastfeeding.

#### Multidimensional Health Locus of Control

Health locus of control (HLoC) was measured using The Multidimensional Health Locus of Control scale (MHLC-Form A) created by Wallston et al. (1978). The scale comprises 18 items and assesses three independent dimensions (each of which contains six items) of HLoC. The Internal Health Locus of Control (IHLoC) dimension measures the degree to which an individual’s health state is believed the result of their individual choices and behaviour (e.g. If I get sick, it is my own behaviour which determines how soon I will get well again). The MHLoC has been used to determine HLoC beliefs in a range of populations (Blondé et al., 2020; Gibek & Sacha, 2019; Güzel et al., 2020; Hildt-Ciupińska et al. 2020; Olagoke et al., 2021), including in Nigeria (Lawal & Idemudia, 2017). The External Chance Health Locus of Control (ECHLoC) dimension subscale measures the strength of an individual’s belief that that their health state is determined by external factors or chance (Wallston et al., 1978). Chance belief is the notion that an individual’s health state is influenced by chance or fate (e.g. My good health is largely a matter of good fortune). External Powerful Others Health Locus of Control (EPOHLoC) refers to the degree to which health is perceived as determined by ‘powerful others’ such as health professionals or other significant people (e.g. Whenever I recover from an illness, it’s usually because other people (for example, doctors, nurses, family, friends) have been taking good care of me). Responses were on a Likert scale ranging from one (strong disagreement) to five (strong agreement). The three subscales have been found to be independent of each other (Holroyd et al., 2017; Wallston et al., 1978). Scores on each of the three subscales were summed separately so that total scores ranged from 6 to 36 with higher scores reflecting stronger beliefs.

### Data Analysis

Data were analyzed using IBM SPSS 22.0 (IBM Corporation, Armonk, New York, USA). The initial data set consisted of 427 cases. Criteria for removing a case were multiple non-response (multiple data missing on more than one measure) and unit non-response (no data on an entire measure). A total of 27 cases were removed leaving a sample of 400 for analysis. IIFAS and HloC scores were checked for normality using Shapiro-Wilk’s test and all were found to have a skew and kurtosis value of below *p* > 0.5. Outliers were checked using boxplots and taking Z scores above 3.29 (Field, 2013), and no outliers were identified. Differences in HLOC subscale scores and IIFAS total scores were calculated between gender using t-tests. Associations between age, IIFAS and HLoC scores were calculated using a Pearson’s correlation. Multiple linear regression analysis was then conducted to determine associations between breastfeeding attitude scores (IIFAS) (dependent variable) and each of the three HLoC subscales (continuous), age (continuous) and gender (independent variable). A second regression model was computed with age excluded since age was not associated with breastfeeding attitudes in the first model. Statistical significance was assumed at *p <* 0.05.

## Results

### Sample Description

Respondents (N = 400) were of a mean age of 34.2 years ranging between 18 and 86 years, of whom 71.3% identified as female. A majority (73.3%) had a university degree. Nearly half were employed (45.5%), self-employed (17%), unemployed (5.5%), students (23.8%) and retired (3.3%). Nearly half (47.5%) were married, 42.5% were single and 9.5% were in ‘other’ relationships. Respondents who identified as single held more positive attitudes to breastfeeding than those in the ‘other (cohabiting)’ category *F* (1, 392) = 2.65, *p* = 0.05, η^2^ = 0.01. Over half of the sample (53.6%) had one or more children, 95% of whom reported having breastfed. Of those who reported breastfeeding a majority (70%) had continued for more than six months with 21.23% having breastfed exclusively for six months. Breastfeeding attitudes did not differ across educational level, occupation, accommodation type or having children.

### The Iowa Infant Feeding Attitude Scale (IIFAS)

Internal reliability of the IIFAS was moderate (α = 0.55). The mean IIFAS score for the entire sample ranged from 36 to 79 (M = 57.67, S.D.= 7.76) (Table 1). Men (M = 58.57, S.D = 7.58) had similar attitudes toward breastfeeding to women (M = 56.57, S.D = 8.06) (*t* (398) = 1.56, *p =* 0.03). Increasing age was associated with lower IIFAS scores (r= -0.12, n = 395, *p* = 0.02).

**Table 1:** Item analysis of the IIFAS (N = 400)

	M	SD	Agree (%)	Neutral (%)	Disagree (%)
1. The nutritional benefits of breast milk last only till the baby is weaned from breast milk.	3.06	1.55	43.8	16.2	40
2. Formula-feeding is more convenient than breast feeding.	3.02	1.37	42	18.2	39.8
3. Breast-feeding increases mother-infant bonding	4.22	1.12	81	10.3	8.8
4. Breast milk is lacking in iron.	3.75	1.16	60.8	27.5	11.8
5. Formula-fed babies are more likely to be overfed than breast fed babies	3.13	1.24	39.5	33.5	27.0
6. Formula-feeding is the better choice if the mother plans to go back to work.	2.66	1.19	24.5	23.5	52.0
7. Mothers who formula-feed miss out on one of the greatest joys of motherhood	3.40	1.28	49.8	25.2	25.0
8. Mothers should not breast-feed in public places such as restaurant.	2.99	1.37	41.0	17.5	41.5
9. Babies fed breast milk are healthier than babies who are formula fed	3.70	1.26	59.0	23.2	17.8
10. Breast fed babies are more likely to be overfed than formula-fed babies.	3.11	1.22	35.0	38.5	26.5
11. Fathers feel left out if a mother breast feeds.	3.18	1.32	43.5	26.0	30.5
12. Breast milk is the ideal food for babies	4.03	1.20	74.0	13.3	12.8
13. Breast milk is more easily digested than formula	3.96	1.21	71.0	16.5	12.5
14. Formula is as healthy for an infant as breast milk.	3.04	1.22	35.0	34.0	31.0
15. Breast feeding is more convenient than formula feeding	3.52	1.29	53.8	20.8	25.5
16. Breast milk is less expensive than formula	4.31	1.04	82.0	9.8	8.3
17. A mother who occasionally drinks alcohol should not breastfeed her baby.	2.09	1.19	11.8	24.8	63.4

Note: Items 1,2,4,6,8,10,11,14,17 were reversed scored. Disagree includes ‘strong disagreement’ and ‘disagreement’. Agree includes ‘strong agreement’ and ‘agreement’

**Table 2:** Iowa infant feeding attitude scale (IIFAS) and health locus of control (HLOC) (N = 400)

	Mean	Std. Deviation
IIFAS	57.67	7.99
Internal HLOC	24.98	4.98
Chance HLOC	20.61	5.49
Powerful Others HLOC	23.36	5.46

### Multidimensional Health Locus of Control Scale (MHLoCs)

Internal reliability of the HLoC dimensions for the current sample was moderate on the IHLoC subscale (0.54), good on the EPHLoC subscale (0.75) and moderate on the ECHLoC subscale (0.59). Overall, internal reliability on all three dimensions was at a level deemed acceptable in health research (Streiner, Norman & Cairney, 2015) and similar to internal reliability values obtained in other studies (Konkolÿ Thege et al., 2014; Hewson & Charlton, 2005; Kuwahara et al., 2004). The mean scores on each subscale for the entire sample were: IHLoC (M = 24.98, S.D = 4.98); EPOLoC (M = 23.36, S.D = 5.46); and, ECHLoC (M = 20.61, S.D = 5.49) (Table 2). Cronbach’s alpha indicated moderate reliability on the IHLoC subscale (0.54), good on the EPHLoC subscale (0.75) and moderate on the ECHLoC subscale (0.59).

Gender differences were found on ECHLoC F(3, 396) = 3.49, *p* = 0.02; Wilk’s Λ = 0.97. Women scored higher than men on ECHLoC F(1, 398) = 9.10; *p* = 0.003; ƞ^2^= 0.02; and EPOHLoC F(1,398) = 5.36, *p* = 0.02; ƞ^2^= 0.013. No gender differences were found for IHLoC F(1, 398) = 2.59; *p* = 0.11; ƞ^2^= 0.001.

Increasing age was associated with higher ECHLoC scores (r = 0.12, n = 395, *p* = 0.01) and EPOHLoC (r = 0.19, n = 395, *p* = 0.01). No significant association was observed between age and IHLoC (IHLoC (r = 0.04, n = 395, *p* = 0.42).

### Iowa Infant Feeding Attitudes Scale (IIFAS) and Multidimensional Health Locus of Control Scale (MHLoCs)

Simple correlations were computed between breastfeeding attitudes and external dimensions of HLoC and all relationships were found to be negative (ECHLoC (r= -0.21, n = 400, *p* = 0.001) and EPHLoC (r= -0.16, n = 400, *p =* 0.001). Correlations indicated no relationship between breastfeeding attitudes and IHLoC (r = 0.07, n = 400, *p* = 0.15).

Multiple linear regression analysis was initially conducted with age (continuous variable), and indices of health locus of control (internal; external powerful others; external chance) entered as explanatory (independent) variables and with breastfeeding attitude (IIFAS) score as the outcome variable. The model significantly explained breastfeeding attitudes (F (4,366) = 6.27, *p* = 0.001) with an R^2^ of 0.064. Higher scores on Internal HLoC were (positively) associated with higher IIFAS scores reflecting more favourable breastfeeding attitudes (*p* = 0.001). Higher scores on External ‘Chance’ HLoC were (negatively) associated with lower IIFAS scores reflecting less favourable breastfeeding attitudes (*p* = 0.001). External ‘Powerful Others’ was not associated with IIFAS scores (*p* = 0.062).

Because age was not significantly associated with HLoC in the initial model (*p* = 0.716), age was excluded from the subsequent multiple linear regression analysis. A second multiple linear regression model was performed to explain breastfeeding attitude (IIFAS) scores (dependent variable) with gender, Internal HLoC, External Chance HLoC and External Powerful Others HLoC as explanatory (independent) variables (Table 2). The revised model was significant (F(4,395) = 8.39, *p* = 0.0001) with an R^2^ of 0.078 and explained 7.8% of the variance in breastfeeding attitude (Table 3). Higher IHLoC was significantly (positively) associated with higher (more favourable attitudes to breastfeeding) IIFAS scores (β = 0.17, t (395) = 3.30, *p* = 0.001). Higher ECHLoC was associated with lower (reflecting less favourable attitudes to breastfeeding) IIFAS scores (β= -0.18, t (395) = -3.26, *p* = 0.01). Higher EPOHLoC was associated with lower (reflecting less favourable attitudes to breastfeeding) IIFAS scores (β= -0.12, t (395) = -2.16, *p* = 0.03). Gender was not associated with IIFAS scores (β= -0.09, t= (395) -1.755, *p* = 0.08).

**Table 3:** Associations between iowa infant feeding attitude scale (IIFAS) and gender, internal health locus of control (HLoC), chance HLoC and powerful others HLoC (N = 400)

	B	Std. Error	β	t	Sig
Constant	62.431	2.566		24.331	0.000
Gender	-1.510	0.860	− 0.086	-1.755	0.080
Internal HLoC	0.276	0.084	0.173	3.301	0.001
Chance HLoC	− 0.262	0.080	− 0.181	-3.262	0.001
Powerful HLoC	− 0.180	0.083	− 0.123	-2.156	0.032

## Discussion

This analysis explored associations between breastfeeding attitudes, gender, age and health locus of control orientation in Nigerian society. As hypothesised, higher IHLoC was associated with more positive attitudes to breastfeeding. As IHLoC increased by one point, IIFAS scores increased 0.28. Also as predicted, higher ECHLoC and EPOHLoC scores were associated with lower (more negative) attitudes to breastfeeding, and according to the model. Health locus of control (HLoC) accounted for 7.8% of the variance in breastfeeding attitude.

That attitudes became more favourable to breastfeeding with increasing IHLoC is in keeping with previous research that has looked at IHLoC and other dietary health related behavior (Jang & Baek, 2018; Rongen et al., 2014; Steptoe & Wardle, 2001). Assuming breastfeeding attitudes are linked to behaviour, the findings are also consistent with other studies which have found that individuals with high IHLoC were more likely to intend to breastfeed (Haslam et al., 2003). People who are lower in IHLoC, therefore, may need specific intervention to motivate breastfeeding (Marton et al., 2020; Davey et al., 2019). Breastfeeding promotion messages should be conveyed in a way that engenders greater internal locus of control and seek to give people confidence in their ability to deal with problems encountered in exclusively breastfeeding.

Also as predicted, high EPHOLoC was associated with less favourable attitudes toward breastfeeding. With every point increase in EPHOLoC a 0.18 decrease in IIFAS score was observed. This finding agrees with other research (Steptoe & Wardle, 2001) indicating that EPOHLoC can be associated with less healthy dietary practices. A high EPOHLoC implies that individuals believe that health is determined by others perceived as powerful. The finding that less favourable attitudes to breastfeeding were associated with greater EPOHLoC may go some way towards understanding why initiatives to promote exclusive breastfeeding have achieved limited success in Nigeria (Agho et al., 2019). Breastfeeding promotion should identify and target these groups in breastfeeding awareness programs, possibly through intermediaries perceived to be ‘powerful’ with a view to engendering positive attitudes toward breastfeeding.

As expected, individuals who scored high on ECHLoC exhibited less favourable attitudes towards breastfeeding. This agrees with results of previous studies indicating that individuals who scored high on ECHLoC were less likely to engage in health promoting behaviour (Grotz et al., 2011) or eat healthily (Helmer et al., 2012). That individuals who scored high on ECHLoC had less favourable attitudes towards breastfeeding implies that infant health is perceived a function of chance rather than how they are fed. Breastfeeding promotion should seek to counter fatalist views by providing information on benefits of breastfeeding.

Contrary to prediction and previous surveys that report more positive attitudes to breastfeeding in women (Jefferson, 2017; Chang et al., 2012; Mitchell-Box et al., 2013), the regression model indicated that the association between breastfeeding attitudes and health locus of control did not differ by gender. Also, contrary to hypothesis, age did not contribute to the relationship between HLoC and breastfeeding attitudes, a finding that appears to contradict evidence that younger women in Nigeria are less likely than older women to breastfeed exclusively (Olasinde et al., 2021; Emmanuel & Clow, 2020). Evidence for age differences in breastfeeding attitudes, however, appears inconsistent. Although some previous studies have also reported null findings regarding age (Cotelo et al., 2018; Ishak et al., 2014), others found more favourable attitudes to breastfeeding in younger people (Charafeddine et al., 2016), or more positive attitudes towards breastfeeding with increasing age (Lau et al., 2017; 2016). These results imply that efforts to improve attitudes to breastfeeding in Nigeria should target men and women and different age groups similarly.

Although the model strength was relatively weak, the results are consistent with the findings of other studies on HLoC (Cheng et al., 2016). Although mean scores on IHLoC and EPOHLoC were similar to figures originally reported by Wallston and colleagues (1978), ECHLoC scores were somewhat higher (23.36 in the present study vs. 16.2). This difference may be attributed to disparities in sampling and cultural background as Wallston and colleagues (1978) sample were students and air passengers recruited in the United States. That the questionnaire employed well validated scales and that the internal reliability of subscales was fairly good (Wallston, 2005) strengthens the results. Meanwhile, the results of the present study suggest that the sample are relatively higher in external HLoC which may render them difficult to reach through conventional breastfeeding promotion campaigns.

A potential limitation to consider when interpreting these data is that variables were self-reported and as such, subject to the constraints of memory. Convenience sampling resulted in a larger proportion of women in the sample and a bias toward people who spent longer in education. Given women who attain higher education levels are more likely to breastfeed (Alkusayer et al., 2018a; Wilkins et al., 2012), the outcomes of this study may not be generalizable to the general populace of Nigeria.

## Conclusion

This study is novel in focusing upon societal attitudes to breastfeeding and health locus of control in Nigeria and indicates a need to adjust breastfeeding promotion interventions to account for individual and societal HLoC (Marton et al., 2020; Jang & Baek, 2018). Designing societal interventions to promote breastfeeding that take HLoC beliefs into account may hold the key to improving exclusive breastfeeding rates as advocated by the WHO (2018). HLoC may need to be considered in efforts to promote more positive attitudes to breastfeeding. That people who were lower in internal HLoC had more positive attitudes toward breastfeeding and people with higher external HLoC held more negative attitudes implies a need to target breastfeeding promotion toward HLoC orientation.

## Data Availability

The data set is available on request and for publication.
